# Molecular profiling of appendiceal serrated lesions, polyps and mucinous neoplasms: a single-centre experience

**DOI:** 10.1007/s00432-021-03589-4

**Published:** 2021-03-12

**Authors:** Giada Munari, Gianluca Businello, Paola Mattiolo, Gianmaria Pennelli, Marta Sbaraglia, Chiara Borga, Salvatore Pucciarelli, Gaya Spolverato, Claudia Mescoli, Francesca Galuppini, Antonio Sommariva, Elena Bellan, Sara Lonardi, Fotios Loupakis, Claudio Luchini, Angelo Paolo Dei Tos, Matteo Fassan

**Affiliations:** 1 Veneto Institute of Oncology, IOV-IRCCS, Padua, Italy; 2 Surgical Pathology and Cytopathology Unit, Department of Medicine (DIMED), University of Padua, Via Gabelli 61, 35121 Padua, Italy; 3 Department of Diagnostics and Public Health, Section of Pathology, University and Hospital Trust of Verona, Verona, Italy; 4 Department of Surgical, Oncological, and Gastroenterological Sciences, Section of Surgery, University of Padua, Padua, Italy; 5 Advanced Surgical Oncology Unit, Surgical Oncology of the Esophagus and Digestive Tract, Veneto Institute of Oncology IOV-IRCCS, Padua, Italy; 6 First Oncology Unit, Veneto Institute of Oncology, IOV-IRCCS, Padua, Italy

**Keywords:** Appendiceal neoplasms, Serrated lesions, Molecular pathology, Biomarkers, *Pseudomyxoma peritonei*

## Abstract

**Purpose:**

Non-neuroendocrine neoplasms of the appendix are a phenotypically heterogeneous group of lesions; a comprehensive molecular characterization of these tumors is still lacking.

**Methods:**

A total of 52 samples taken from 49 patients was evaluated: 18 sessile serrated lesions (SSL; 3 with dysplasia), 2 high-grade tubular adenomas, 1 tubulo-villous adenoma,1 hyperplastic polyp, 18 low-grade appendiceal mucinous neoplasms (LAMN), 3 high-grade appendiceal mucinous neoplasms (HAMN) and 9 mucinous adenocarcinomas. Hotspot mutational profiling of the *RNF43, SMAD4*, *KRAS*, *NRAS*, *BRAF* and *PIK3CA* genes was performed. Expression of p53, MLH1, PMS2, MSH2, and MSH6 was evaluated by immunohistochemistry.

**Results:**

*KRAS* was the most frequently mutated gene (53.9% of cases), followed by *RNF43* (15.4%), and *BRAF* (13.5%). In particular: *KRAS* was mutated in 44.4% of adenocarcinomas, 66.7% of HAMNs, 61.1% of LAMNs, 53.3% of SSL without dysplasia and in 66.7% of SSL with dysplasia; *RNF43* was mutated in 33.3% of adenocarcinomas, 66.7% of HAMNs, 11.1% of LAMNs and in 6.7% of SSL without dysplasia; *BRAF* was mutated in 11.1% of adenocarcinomas, 26.7% of SSL without dysplasia and in 5.6% of LAMNs. Only a case of high-grade tubular adenoma showed mismatch repair deficiency, while immunohistochemical expression of p53 was altered in 21.1% of cases.

**Conclusions:**

The histological phenotypic similarities between appendicular mucinous lesions and serrated colon lesions do not reflect a similar genetic landscape. Mismatch repair deficiency is a rare event during appendiceal mucinous carcinogenesis.

## Introduction

Primary tumours of the appendix are reported in less than 1% of appendectomy specimens (Connor et al. 1998; Smeenk et al. 2008; WHO Classification of Tumours Editorial Board 2019).

Among the others, the appendicular epithelial non-neuroendocrine neoplasms are a heterogeneous group of lesions, that the Peritoneal Surface Oncology Group International (PSOGI) have proposed to classify in two main groups: non-invasive and invasive neoplasms (Carr et al. 2016). Sessile serrated lesions with or without dysplasia (SSLd/SSL), conventional adenoma resembling colorectal type, low-grade appendiceal mucinous neoplasm (LAMN), and high-grade appendiceal mucinous neoplasm (HAMN) belong to the non-invasive group; while mucinous adenocarcinoma, mucinous adenocarcinoma with signet ring cells, signet ring cell carcinoma and non-mucinous carcinoma are considered invasive neoplasms (Carr et al. 2016). All these neoplasms have the common potential to cause *pseudomyxoma peritonei* (PMP). PMP is a clinical entity characterized by grossly evident, diffuse, intra-abdominal mucinous ascites and by peritoneal implants (Carr et al. 2016).

Early-stage appendicular neoplasms can be treated with surgery and long-term benefits have also been achieved with cytoreductive surgery and heated intraperitoneal chemotherapy (HIPEC) (Reghunathan et al. 2018). On the contrary, neither a standard of care nor specific targeted therapies currently exist for patients with advanced or unresectable tumours. Despite the lack of consensus, chemotherapeutic regimens approved for colorectal cancer (CRC) are most commonly used for advanced cases (Tokunaga et al. 2019; Pietrantonio et al. 2014).

Disseminated appendiceal mucinous neoplasms exhibit a spectrum of clinical behaviours, ranging from slow-growing tumours with considerable risk of recurrence and eventual death, to highly aggressive neoplasms with a high risk of early death (Carr et al. 2012; Davison et al. 2014).

Although in the last few years an increasing attention has been paid to improve the classification and definition of appendicular neoplasms, a deeper molecular characterization of these tumors is still lacking.

## Materials and methods

### Patients and tumor samples

**Fig. 1:**
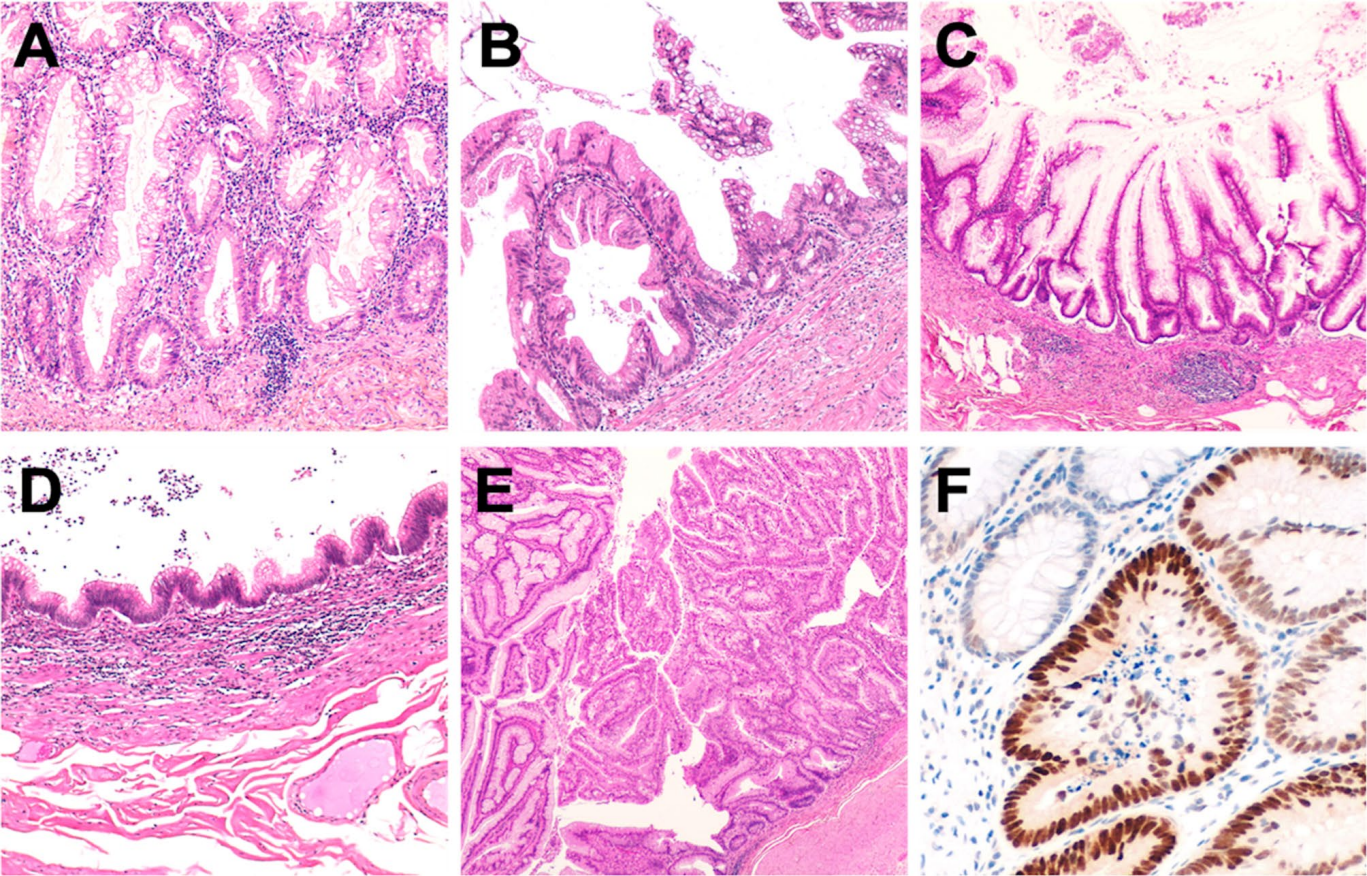
Representative examples of the considered series of appendiceal mucinous neoplasms. **a** A serrated lesion characterized by serrated crypt profiles. **b** Transition phase from a serrated lesion with dysplasia (left) to a low-grade mucinous neoplasm (right). **c**, **d** Different phenotypic appearance of LAMN. **e** HAMN showing a complex pattern of growth. **f** p53 immunohistochemical expression of the neoplastic glands of the lesion showed in **e**. Original magnifications ×10, ×20 and ×40

A total of 52 appendiceal lesions from 49 Caucasian patients (F/M = 26/24; age 71.95 ± 13.3) were collected from 2011 to 2018 in the Department of Surgical Pathology, University of Padua Medical Centre*.* Pathology reports and hospital charts were reviewed to collect the following information: type of surgical procedure, age at presentation, gender, and anatomic extent of tumour at diagnosis. All information regarding human material was managed using anonymous numerical codes, and all samples were handled in compliance with the Declaration of Helsinki. The 52 appendiceal lesions were jointly re-evaluated by three gastrointestinal pathologists according to 2019 WHO classification of tumours of the digestive system (WHO Classification of Tumours Editorial Board 2019) and classified as: 18 serrated lesions (3 with dysplasia), 2 high-grade tubular adenomas, 1 tubulo-villous adenoma, 1 hyperplastic polyp, 18 low-grade appendiceal mucinous neoplasms (LAMN), 3 high-grade appendiceal mucinous neoplasms (HAMN) and 9 mucinous adenocarcinomas (no signet-ring cell adenocarcinoma was considered; Fig. 1)*.*

Neoplastic areas were manually microdissected from 10 μm unstained histological sections formalin-fixed and paraffin-embedded (FFPE) samples using the original haematoxylin and eosin-stained slide as guide to select the region of interest. The DNA was extracted from each target using the QIAamp DNA FFPE tissue kit (Qiagen, Milan, Italy) according to the manufacturer’s instructions.

### RNF43 and SMAD4 mutational analysis

The DNA isolated from tumour samples was used to perform PCR amplification of *RNF43* (exons 2, 3, 4, 5, 6, 7 and 8) and *SMAD4* (exons 1, 2 and 3) genes. Amplified PCR products were purified and sequenced by the Sanger method. Primers used for PCR and Sanger sequencing are available upon request.

### Hotspot KRAS, NRAS, BRAF and PIK3CA genes mutational profiling

The mutational status of *KRAS, NRAS, BRAF and PIK3CA* hotspot regions was assessed using the high-throughput genotyping platform Sequenom MassARRAY System (Agena Biosciences, San Diego, CA, USA) and the Myriapod Colon Status Kit (Diatech Pharmacogenetics, Jesi, Italy), following the manufacturer’s instructions. This molecular array allows to identify the most relevant mutations of *KRAS* (codons 12, 13, 59, 61, 117, and 146), *NRAS* (codons 12, 13, 18, 59, 61, 117, and 146), *BRAF* (codons 594, 600, and 601), and *PIK3CA* genes (codons 38, 81, 88, 93, 108, 118, 345, 420, 539, 542, 545, 546, 549, 1021, 1025, 1043, 1047, and 1049).

### Immunohistochemical analysis

Immunohistochemical staining for p53 (clone DO-7; Agilent, Santa Clara, CA; prediluited), MLH1 (clone ES05; Agilen; dilution 1:25), PMS2 (clone EP51; Agilent; dilution 1:20); MSH2 (clone FE11; Agilent; dilution 1:50), and MSH6 (clone EP49; Agilent; dilution 1:25) was performed on a Leica Bond system (Bond-III; Leica Microsystems, Buccinasco, Milan, Italy). The slides were jointly assessed by two pathologists. Nuclear immunostaining for MLH1, PMS2, MSH2, and MSH6 was evaluated following the AIFEG-SIAPeC criteria to identify mismatch repair-deficiency (MMRd) and mismatch repair-proficiency (MMRp) (Remo et al. 2016). Expression of p53 was considered as “altered” in the presence of more than 30% neoplastic nuclei showing a strong staining or in the case of complete absence in the context of a positive background.

## Results

### Clinico-pathological features of the series

Samples obtained from 49 Caucasian patients were included in the study. Specifically, appendectomy was performed for PMP in 2 cases, during CRC surgery in 32 cases, abdominal surgery for extra-colic neoplastic diseases in 5 cases (1 IPMN, 1 uterine adenocarcinoma, 1 small bowel adenocarcinoma and 2 gallbladder cancer). For 3 cases, patients underwent appendectomy secondary to bowel surgery for non-neoplastic disease (i.e. ileal resection due to umbilical hernia, treatment of not-responsive ulcerative colitis, anastomotic dehiscence). Seven patients required appendiceal removal because of appendiceal pathology (1 mucocele, 1 urgent intervention for intestinal obstruction and 5 acute appendicitis).

For three cases, two separate lesions were microdissected from the same appendix: the first case had the concomitant presence of a hyperplastic polyp (HP) and a tubulo-villous adenoma; in the second case, the coexisting LAMN and HAMN features were microdissected and analysed separately; in the third case, the mucinous adenocarcinoma with its adjacent high-grade tubular adenoma were microdissected separately.

### Molecular landscape of appendicular lesions (Fig. 2)

**Fig. 2:**
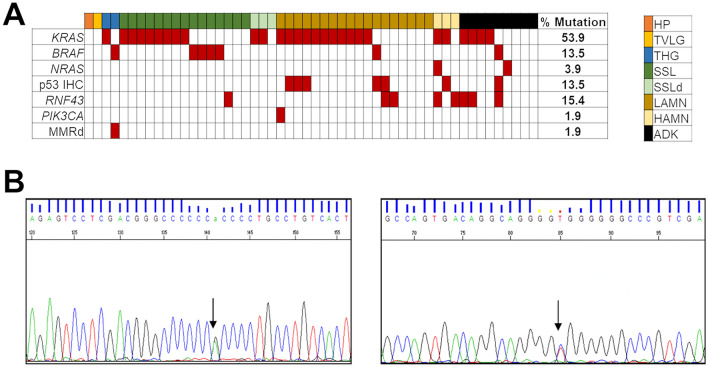
a Graphic representation of the results of the molecular profiling of mucinous appendicular neoplasia. On the top, horizontally, the type of lesion is reported (colors legend on the right). Vertically, on the left, the names of mutated/altered genes (italics, capital letters) or proteins with altered expression is shown. Alterations are indicated by red squares. The prevalence of molecular alterations is displayed on the right. *HP* hyperplastic polyp, *TVLG* tubulo-villous adenoma with low-grade dysplasia, *THG* tubular adenoma with high-grade dysplasia, *SSL* sessile serrated lesion, *SSLd* sessile serrated lesion with dysplasia, *LAMN* low grade appendiceal mucinous neoplasm, *HAMN* high grade appendiceal mucinous neoplasm, *ADK* mucinous adenocarcinoma, *MMRd* DNA mismatch repair deficiency. b Representative examples of Sanger sequencing for a mutation identified in the *RNF43* gene. Left electropherogram with the representation of the p.R117H mutated forward sequence of the *RNF43* gene and its respective reverse sequence (bottom right)

#### Matched lesions

Of the three matched samples, the patient carrying concomitant HP and tubulo-villous adenoma with low-grade dysplasia; showed absence of mutations in the genes analysed, normal p53 expression and non-altered microsatellite status. In the second patient, affected by simultaneous LAMN and HAMN, both samples revealed a common driver *KRAS* p.G12D point mutation and the altered p53 expression. Only data on the HAMN lesion were retained for mutational prevalence and Fig. 2 output. The third case included a mucinous adenocarcinoma and the tubular adenoma from which the carcinoma initiated; both lesions resulted wild type for the tested genes, except for a p.G12V point mutation in the *KRAS* gene.

#### Appendiceal mucinous adenocarcinomas

Our results showed that *KRAS* was the main mutated gene in appendiceal mucinous adenocarcinoma, especially in exon 2 (4/9 cases), followed by *RNF43* (3/9). One case of appendiceal mucinous adenocarcinoma had *BRAF* p.V600E mutation and alteration in the *RNF43* gene with stable expression of MMR proteins. This was the only case showing strong IHC positivity for p53. *NRAS* gene was mutated in one case.

#### High-grade appendiceal mucinous neoplasms (HAMN)

Two (66.67%) out of 3 cases had codon 12 of exon 2 *KRAS* gene mutation, and one of them had p53 dysregulation.

Two out of three cases had alterations in the *RNF43* gene. The first had an alteration in *RNF43* p.R296C in concomitance with the mutations in *KRAS* and *NRAS*; the second case showed the *RNF43* p.R117H mutation and p53 dysregulation with wild type *BRAF/RAS* genes.

#### Low-grade appendiceal mucinous neoplasms (LAMN)

The majority of LAMNs showed alterations in *KRAS*: 55.56% (10/18) in exon 2 and 5.55% (1/18) in exon 4. One case had a mutation in *BRAF* p.V600E (5.6%). Another case showed the concurrent presence of mutations in *KRAS* and *BRAF*. In addition, 4/18 LAMN were wild type for the entire gene panel. An altered p53 IHC expression was observed in five lesions.

#### Serrated sessile lesions (SSL) and serrated lesions with dysplasia (SSLd)

8/15 (53.33%) SSL harboured *KRAS* main gene mutations: 6/15 cases (40%) in exon 2 and 2/15 cases (13.33%) in the exons 3 and 4. Furthermore, 4/15 cases (26.67%) harboured *BRAF* p.V600E mutation. Three cases were SSLd; 2/3 (66.67%) had exon 2 *KRAS* mutation; 1/3 (33.33%) was *BRAF/RAS* wild type. One case showed the simultaneous presence of mutations in *KRAS* and *RNF43*.

An altered IHC p53 expression was observed in three SSL lesions. One SSLd also showed strong IHC positivity for p53.

One case resulted mutated in exon 5 of the *RNF43* gene. One case of SSL was *RNF43* p.C222Y mutated and alteration in p53 expression was detected by IHC.

#### Other lesions

**Fig. 3:**
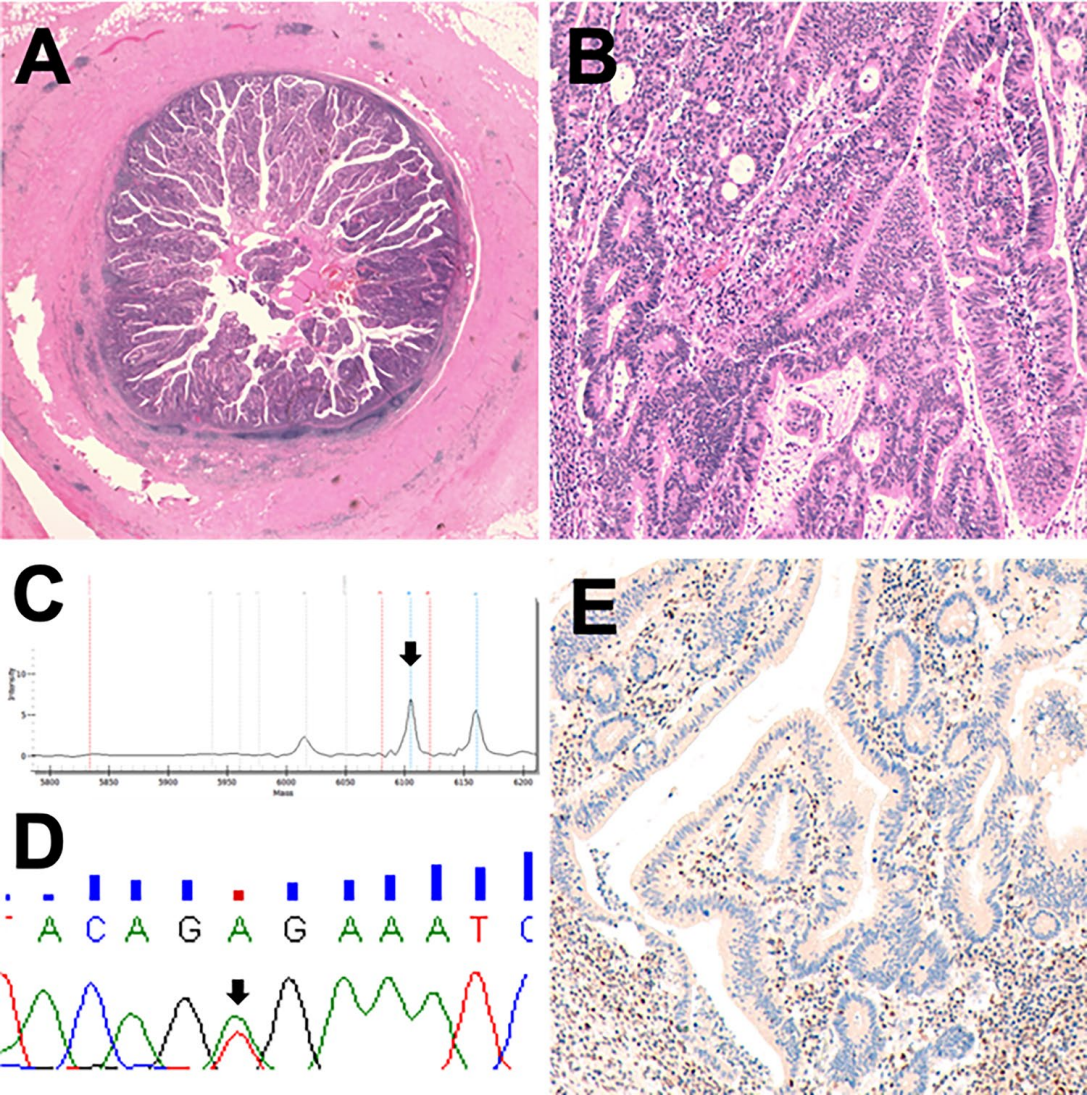
A tubular adenoma with high-grade dysplasia showing a peculiar molecular profile for appendicular mucinous neoplasm. **a** Gross characteristics of the lesion. **b** High grade dysplastic glands. **c**, **d** Representative Sequenom MassArray output profile and Sanger chromatogram of the p.V600E *BRAF* mutation. **e** Loss of PMS2 in the epithelial neoplastic cells. Of note, this was the only case with MMRd of the entire series

The tubular adenoma with high-grade dysplasia was characterized by a *BRAF* p.V600E mutation and mismatch repair deficiency (loss of MLH1/PMS2) (Fig. 3). Of note, this was the only case of microsatellite instability found in the entire series analysed. The tubulo-villous adenoma resulted wild type for all genes investigated. The only HP was wild type for all the tested genes, MMRp and with retained p53 expression.

## Discussion

This study molecularly profiled a relatively large spectrum of preinvasive and mucinous neoplasms of the appendix.

Consistently with the literature, *KRAS* mutations predominated among the different appendiceal lesions: in fact, half of our cases showed *KRAS* point mutations and these mutations harboured in SSLs (about 60%) as well as in adenocarcinomas (44%). *KRAS* mutations (preferentially observed in exon 2) seem to have a pivotal role in the development of appendiceal mucinous neoplasms (Kabbani et al. 2002; Borazanci et al. 2017). *KRAS* is mutated in 41–100% of appendiceal mucinous adenomas (Szych et al. 1999; Zauber et al. 2011; Yantiss et al. 2007; Tsai et al. 2019; Liao et al. 2020; Yanai et al. 2020). Pai et al. (2014) analysed a series of 132 appendiceal lesions, revealing that serrated lesions of the appendix often harbour *KRAS* mutations and only infrequently display *BRAF* mutations. Moreover, *KRAS* mutations have been identified in a high proportion of disseminated mucinous neoplasms (Tokunaga et al. 2019; Davison et al. 2014; Szych et al. 1999; Zauber et al. 2011; Tsai et al. 2019). Notably, Davison and colleagues (Davison et al. 2014) have reported that, in their series, none of the high-grade mucinous adenocarcinomas with a predominant signet ring cell component had *KRAS* mutation.

All these findings support the idea that *KRAS* may be biologically more important than *BRAF* in appendiceal lesions, and the serrated pathway of carcinogenesis may have less relevance in the appendix than in the colon (WHO Classification of Tumours Editorial Board 2019; Carr et al. 2009; Murakami et al. 2018). We have detected *BRAF* mutations in 13% of our cases. Our findings are quite different from previous data reported in the literature, which pinpointed a relative rarity of *BRAF* mutations in appendicular SSLs (Pai et al. 2014), with the exception of Tsai and colleagues (Tsai et al. 2019) that found 78%(7/9) of serrated polyps characterized by a *BRAF* p.V600E mutation. *KRAS* and *BRAF* mutations are mutually exclusive in CRC: the shortage of *BRAF* mutations detected in appendiceal neoplasms with PMP seems to be a consistent consequent high prevalence of *KRAS* mutations among appendiceal mucinous neoplasms (Tokunaga et al. 2019).

Another frequently mutated gene in appendiceal tumours is *GNAS* (Ang et al. 2018; Alakus et al. 2014), which was not analysed in our series. Curiously, *GNAS* mutations coexist with the *KRAS* mutation in 65–85% of cases (Gleeson et al. 2018; Stein et al. 2020). Liu and colleagues (Liu et al. 2014) reported *KRAS* and *GNAS* are the most commonly mutated genes in LAMNs and low-grade mucinous adenocarcinomas with PMP. According to Ang and colleagues (Ang et al. 2018), *GNAS* mutations are usually absent in high-grade neoplasms, while mutations in *TP53* are usually absent in low-grade ones. These findings outline a significant association of *GNAS* mutation with low-grade tumours and of *TP53* mutation with high-grade tumours, suggesting the majority of high-grade appendiceal tumours occur de novo, rather than progressing from low-grade neoplasms (Ang et al. 2018; Alakus et al. 2014). Conversely, Hara and colleagues (Hara et al. 2015) reported that LAMNs and appendiceal mucinous carcinomas might share a mutational spectrum comprising *KRAS*, *TP53* and *GNAS* genes, suggesting that mucinous carcinomas might evolve from LAMNs. However, Singhi and colleagues (Singhi et al. 2014) showed that *GNAS* is commonly mutated in both low-grade and high-grade disseminated appendiceal mucinous neoplasms, suggesting that *GNAS* mutation status is not related to the grade of appendiceal mucinous neoplasms. Similarly, Liao and colleagues (Liao et al. 2020) recently showed that HAMN and LAMN share high rates of *KRAS* and *GNAS* co-mutations supporting a common histogenesis and distinguishing them from mucinous adenocarcinoma, which is characterized by *KRAS* mutations in the absence of *GNAS* alterations.

*RNF43* mutations were present in 7 cases (13%), particularly in 3 adenocarcinomas, 2 LAMNs and 2 HAMN. These data support an important role of this mutation in appendiceal neoplasms progression. This seem to be in agreement with previously published data, since alterations of *RNF43* affect both up-stream (Koo et al. 2012) and down-stream (Loregger et al. 2015) activation of Frizzled and consequently of Wnt/beta-catenin pathway (Borowsky et al. 2018; Hao et al. 2016). Yanai and colleagues (Yanai et al. 2020) recently suggested *RNF43* mutations may occur at a later stage of mucinous adenocarcinoma development and may not be associated with PMP. The significance of *RNF43* mutation in appendiceal tumours has not been clarified yet, but its role has been better characterized in IPMN (Lee et al. 2016). This is not the only mutation that connects pancreatic and appendicular neoplasia: indeed, *KRAS* and GNAS mutations have been found in both lesions, suggesting similarities in these two cancerogenic pathways (Lee et al. 2016; Reid et al. 2014; Fischer and Wood 2018; Sakamoto et al. 2015). Similarly, this could explain why abnormal p53 expression by immunohistochemistry is reported in only 21% of our cases. Moreover, prominent mucin production is a common phenotypic feature of LAMN and IPMN (Nishikawa et al. 2013).

Several studies (Valasek and Pai 2018; Nummela et al. 2015) have identified abnormal *TP53* expression in a significant fraction (30%) of high-grade mucinous adenocarcinoma. Considering that only 7% of low-grade tumours show this characteristic, *TP53* status appears to be the only marker associated with the acquisition of an aggressive phenotype in tumours with PMP. We reported an altered immunohistochemical expression of p53 in 13.5% of our cases.

The role of DNA mismatch repair (MMR) in appendix mucosa tumours remains elusive. To the best of our knowledge, only a single study showed a significant prevalence of microsatellite instability in appendicular neoplastic pathology, but most of the tumours tested were characterized by a non-mucinous histotype (Taggart et al. 2013). Moreover, Tokunaga and colleagues (Tokunaga et al. 2019) suggested the immune profile of appendiceal adenocarcinomas is similar to left-sided CRCs but not to right-sided CRCs. Coherently with the literature (Misdraji et al. 2004), genes that code for proteins responsible for the maintenance of microsatellite stability (i.e. expression of the MMR proteins) are not affected in appendicular carcinogenesis: MMR proteins are fully expressed in mucinous neoplasm of the appendix, independently of the histological subtype. This fact suggests that microsatellite instability has no (or minimal) etiological role in the development of appendicular mucinous tumours. In our study, only one case showed MMRd and it was characterized by a V600E *BRAF* mutation, as observed in sporadic MMRd CRCs.

In conclusion, despite the similar phenotype, many non-invasive appendicular mucinous lesions are characterized by a different genetic landscape in comparison to colorectal serrated lesions. The DNA mismatch repair complex seems not to be altered within appendiceal mucinous carcinogenesis.

## Data Availability

Upon request.
